# *Lactobacillaceae* and Cell Adhesion: Genomic and Functional Screening

**DOI:** 10.1371/journal.pone.0038034

**Published:** 2012-05-31

**Authors:** Williams Turpin, Christèle Humblot, Marie-Louise Noordine, Muriel Thomas, Jean-Pierre Guyot

**Affiliations:** 1 IRD, UMR Nutripass, IRD/Montpellier2/Montpellier1, Montpellier, France; 2 INRA, UMR1319, Micalis, “Commensal and Probiotics-Host Interactions” Team, Jouy-en-Josas, France; Indian Institute of Science, India

## Abstract

The analysis of collections of lactic acid bacteria (LAB) from traditional fermented plant foods in tropical countries may enable the detection of LAB with interesting properties. Binding capacity is often the main criterion used to investigate the probiotic characteristics of bacteria. In this study, we focused on a collection of 163 *Lactobacillaceace* comprising 156 bacteria isolated from traditional amylaceous fermented foods and seven strains taken from a collection and used as controls. The collection had a series of analyses to assess binding potential for the selection of new probiotic candidates. The presence/absence of 14 genes involved in binding to the gastrointestinal tract was assessed. This enabled the detection of all the housekeeping genes (*ef-Tu*, *eno, gap, groEl* and *srtA*) in the entire collection, of some of the other genes (*apf, cnb, fpbA, mapA, mub*) in 86% to 100% of LAB, and of the other genes (*cbsA, gtf, msa, slpA*) in 0% to 8% of LAB. Most of the bacteria isolated from traditional fermented foods exhibited a genetic profile favorable for their binding to the gastrointestinal tract. We selected 30 strains with different genetic profiles to test their binding ability to non-mucus (HT29) and mucus secreting (HT29-MTX) cell lines as well as their ability to degrade mucus. Assays on both lines revealed high variability in binding properties among the LAB, depending on the cell model used. Finally, we investigated if their binding ability was linked to tighter cross-talk between bacteria and eukaryotic cells by measuring the expression of bacterial genes and of the eukaryotic MUC2 gene. Results showed that wild LAB from tropical amylaceous fermented food had a much higher binding capacity than the two LAB currently known to be probiotics. However their adhesion was not linked to any particular genetic equipment.

## Introduction

Lactic acid bacteria (LAB) are common inhabitants of a wide variety of environments including the mucosal surfaces of humans and animals and food environments made of milk, plants, and meats. Many strains have been used in the bioprocessing of foods, particularly dairy products. Some are also known as probiotic organisms with a wide range of health promoting effects. Probiotic functionality is well documented for many characters such as the mitigation of lactose intolerance [1], [2], [3]. But supplementary data are needed on immunomodulation, resistance to acid and bile, production of bacteriocins, and adhesion to the intestinal tract [4], [5] to establish a link between consumption of fermented foods and health benefits.

The binding of probiotic bacteria to intestinal cells is expected to have lasting beneficial effects for health including the exclusion of pathogens, immunomodulation and the production of beneficial bacterial molecules [6]. Binding is thus generally considered to be an important property, and, along with survival, is often the main feature investigated in relation with the probiotic characteristics of bacteria. In the last decade, the increasing amount of data dealing with the molecular origin of adhesion has improved our understanding of binding mechanisms. Proteins involved in this mechanism can be separated into five classes: anchorless housekeeping proteins, surface layer proteins, LPXTG-motif proteins, transporter proteins and ‘other’ proteins [7]. To our knowledge, at least 20 genes are reported to be functionally important in the binding of *Lactobacillaceae* to the digestive tract, a third of which were described only recently. In this work, we performed a series of analyses of a collection of 162 LAB strains to assess their binding potential as part of the selection of new probiotic candidates.

The intestine is made up of two main differentiated cell populations, absorptive cells (80%) and secretive cells (4% to 16%), like goblet cells, which are responsible for the secretion of mucus gel [8]. The mucus layer is composed of a mixture of highly glycosylated proteins called mucins that act as a protective barrier against attacks by bile salts, toxins, and pollutants, and that inhibit the binding of bacteria [9], [10], [11]. Many studies have dealt with the adhesion properties of *Lactobacillus* to the intestinal tract, but they mainly used Caco-2 or HT29 cell lines that only mimic enterocytes, thereby underestimating the role of the mucus layer. The use of mucus producing cell lines such as HT29-MTX [12] in addition to traditional HT29 cells lines, is probably a more appropriate way of studying the binding mechanism in relation to the importance of the mucus layer.

Advances in our knowledge of the genetic diversity of LAB and the increasing number of sequenced LAB genomes mean that the functional properties of LAB strains can be studied at both molecular and functional levels. Consequently, in the present study, we screened 14 genes involved in cell binding for which at least one functional analysis had already been performed. We focused on a collection of 163 *Lactobacillaceace* comprising 152 bacteria isolated from a traditional African pearl millet based fermented slurry (*ben-saalga*) [13], four strains isolated from other traditional amylaceous fermented foods, and seven strains from a collection, used as controls. As niche specific adaptation has played a central role in the evolution of LAB [14], the analysis of collections of bacteria from traditional fermented plant foods in tropical countries may enable the detection of LAB with interesting properties. This collection has undergone a series of analyses to assess the strains' binding potential as part of the selection of new probiotic candidates. To investigate possible links between genetic equipment and the binding function, the binding ability of a subset of 30 LAB with different genetic equipment was assessed in mucus producing cell lines (HT29-MTX) and in non-mucus producing cell lines (HT29). The expression of these genes in the LAB after adhesion to the cell lines was also investigated by semi-quantitative real time PCR in three strains whose adhesion capacities differed from those of HT29 and HT29-MTX.

## Results

### Primer design

Among the 14 genes selected because of their involvement in binding mechanisms, seven, *ef-Tu*, *eno, gap*, *groEl*, *srtA, apf,* and *fpbA,* shared conserved regions, thus allowing primers to be designed in several species (Table 1). Conversely, for *cnb, mapA, msa, mub1*, and *mub2* genes, no consensus sequence could be obtained among *Lactobacillaceae*, so primers were designed at species level. For *cbsA*, *gtf* and *slpA* genes, no sequences were available for the bacterial species in our collection, so primers were designed using other LAB species whose sequences were available. For genes annotated as cell surface protein precursors containing MucBP domains, due to the high variability of their sequences, primers were designed on mucus binding domains from different genetic loci. All primers produced amplicons of the desired size with a single band on the agarose gel. Positive controls were done by testing the primers on the DNA from reference strains containing the target genes.

**Table 1:** Primers used to detect the presence or to measure the expression of LAB genes involved in binding ability.

Functions	Gene	Predicted function	Primer sequence 5′to 3′	Primer reference	Melting temperature used (°C)	qPCR efficiency (%)	Species used for the primer design	Article concerned
Housekeeping genes	*ef-Tu*	elongation factor Tu	F_ TCGATGCTGCTCCAGAAGAAA R_ TGGCATAGGACCATCAGTTGC	This study	57.6	60	*L. johnsonii,* *L. helveticus,* *L. acidophilus,* *L. delbrueckii,* *L. reuteri,* *L. salivarius,* *L. fermentum,* *L. casei,* *Leuconostoc mesenteroides,* *P. acidilactici,* *L. oris,* *L. gasseri,* *L. brevis,* *P. pentosaceus,* *L. sakei,* *Lactococcus lactis,* *L. plantarum*	[70]
	*eno*	enolase	F_ CTACCTTGGCGGATTCAACG R_ CGCAAAACCACCTTCGTCAC	This study	59.2	60	*L. fermentum,* *L. plantarum,* *P. pentosaceus,* *L. salivarius*	[52], [71]
	*gap*	glyceraldehyde-3-phosphate dehydrogenase	F_ GTTCTTGAATGTACWGGTTTCTACACT R_ TTCGTTRTCGTACCAAGCAACA	This study	55.0	ND	*L. plantarum,* *P. pentosaceus,* *L. johnsonii,* *L. acidophilus,* *L. delbrueckii,* *L. casei,* *L. crispatus,* *L. helveticus,* *L. reuteri,* *L. brevis,* *L. sakei,* *L. fermentum,* *Lactococcus garvieae,* *Lactococcus lactis,* *L. salivarius L. casei,* *L. gasseri*	[71], [72]
			F_ ACTGAATTAGTTGCTATCTTAGAC R_ GAAAGTAGTACCGATAACATCAGA	[73]	55.0	114	*L. plantarum*	[71], [72]
	*groEl*	heat shock protein 60	F_ TTCCATGGCKTCAGCRATCA R_ GCTAAYCCWGTTGGCATTCG	[13]	58.0	63	*L. salivarius,* *Leuconostoc mesenteroides,* *L. casei,* *L. delbrueckii,* *P. pentosaceus,* *P. acidilactici*	[7]
	*srtA*	sortase	F_ ATGGGGCARGGTAACTACGC R_ GCCCCGGTMTYATCACAGGT	This study	59.2	77	*L. fermentum,* *L. plantarum,* *P. pentosaceus,* *L. salivarius*	[24]
Binding related genes	*apf*	aggregation-promoting factors	F_ YAGCAACACGTTCTTGGTTAGCA R_ GAATCTGGTGGTTCATAYWCAGC	[13]	53.0	57.0	*L. plantarum,* *L. salivarius,* *L. fermentum,* *P. pentosaceus,* *P. acidilactici*	[22]
	*cbsA*	collagen-binding S-layer	F_ TTGGTACTGACAAGGTWACTCGTT R_ TGTCAGCGTTGATGWACTTGC	This study	57.2	ND	*L. crispatus,* *L. gallinarum,* *L. helveticus,* *L. acidophilus,* *L. suntoryeus.*	[74]
	*cnb*	collagen-binding protein	F_ CGTGGAGAAGTCGGTGGATG R_ CATTGCTATGACGCCGGAAC	This study	60.1	59	*L. fermentum*	[11], [12]
	*fpbA*	fibronectin-binding protein	F_ WGCYAAYCGGAAGAATCACC R_ ACCGAGTTCGTYRCGGGTCR	This study	58.0	73	*P. pentosaceus,* *L. fermentum,* *L. salivarius,* *L. plantarum*	[25]
	*gtf*	glucan synthase	F_ ACACGCAGGGCGTTATTTTG R_ GCCACCTTCAACGCTTCGTA	[13]	58.0	ND	*L. diolivorans,* *P. parvulus,* *P. damnosus,* *L. suebicus*	[75]
	*mapA*	mucus adhesion promoting protein	F_ TGGATTCTGCTTGAGGTAAG R_ GACTAGTAATAACGCGACCG	[73]	50.0	57	*L. plantarum*	[76]
	*msa*	mannose-specific adhesin	F_ GCAAGACGCTATCGGGTTCA R_ TAACGCCTGCGACTCTCCTG	This study	59.8	90	*L. plantarum*	[26]
	*mub1*	mucin-binding protein	F_ GTAGTTACTCAGTGACGATCAATG R_ TAATTGTAAAGGTATAATCGGAGG	[73]	50.0	69	*L. plantarum*	[23], [25]
	*mub2*	mucin-binding protein	F_ ACGCGTATTGCGGGTAATGA R_ CGCCCCTGAAGTGGGATAGT	This study	60.0	56	*L. plantarum*	[23], [25]
	*slpA*	surface layer protein	F_ TTGCAGATCCTGTTGTTCCA R_ TGTACTTGCCAGTTGCCTTG	This study	59.9	ND	*L. acidophilus,* *L. helveticus,* *L. crispatus,* *L. suntoryeus,* *L. gallinarum*	[25]

ND: primer pairs were not used for the qPCR assay.

### Detection of genes involved in binding mechanisms

**Figure 1:**
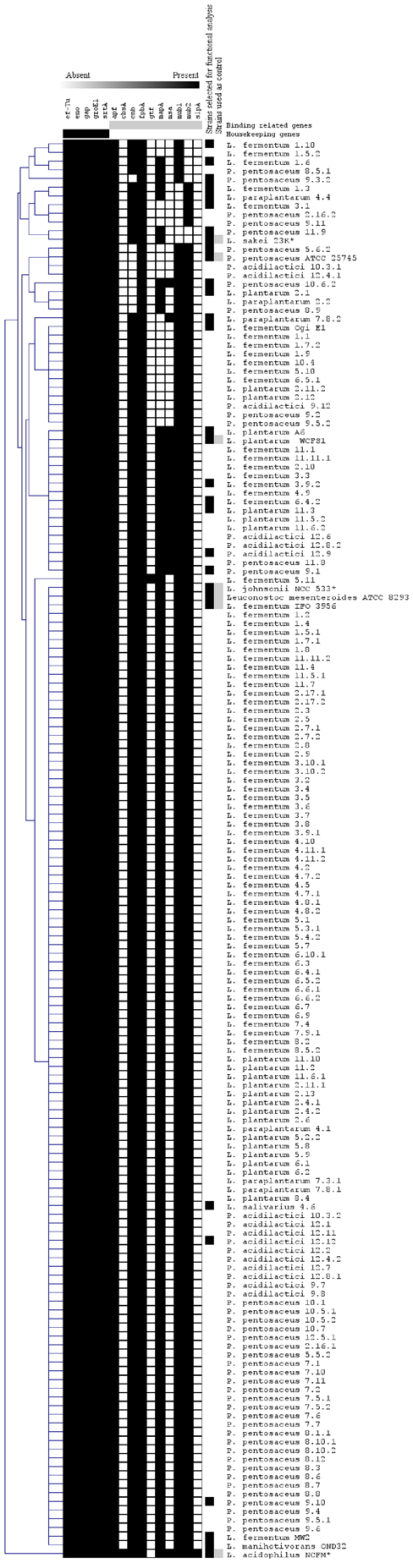
Distribution of genes involved in binding to the gastrointestinal tract in a collection of LAB sampled from starchy fermented foods and in strains used as positive controls. The role of the gene is indicated at the top of the column corresponding to the different strains. The absence of a gene is indicated in white and its presence in black. Sequenced strains are in gray. Strains selected for the adhesion assays and the mucus degrading assays are in black. Genes in *L. sakei* 23K, *L. johnsonii* NCC533, and *L. acidophilus* NCFM were predicted by *in silico* analysis, except for the *cbsA* and *slpA* genes, which were detected on *L. acidophilus* NCFM by PCR. The dendrogram shows estimated relationships among the strains and was constructed by average-linkage hierarchical analysis using Mev 4.4 software [69].

The results of gene detection are presented in figure 1. As expected, all the housekeeping genes (*ef-Tu*, *eno, gap, groEl* and *srtA*) that were also involved in binding mechanisms were found in all LAB. Some of the other genes (*apf, cnb, fpbA, mapA, mub1,* and *mub2*) were detected in 86% to 100% of LAB, whereas others (*cbsA, gtf, msa, slpA*) were found in 0% to 8%. For each gene screened, one amplicon obtained from PCR amplification of DNA extracted from one isolate from each species in the collection was sequenced. At least 91% similarity was found with the corresponding gene in the strains *L. plantarum* JDM1, *L. plantarum* IMAU60049 (13304), *L. plantarum* WCFS1, *L. fermentum* IFO 3956, *P. pentosaceus* ATCC 25745, and *L. salivarius* UCC118 (Accession Number HE609007 to HE609029). Most of the bacteria isolated from the pearl millet slurries had a genetic profile favorable for their binding to the gastrointestinal tract. The distribution of binding related genes was not species-specific, as they were distributed equally among all the isolates of the seven species from the collection.

### Binding assay to HT29 and HT29-MTX cell lines

**Figure 2:**
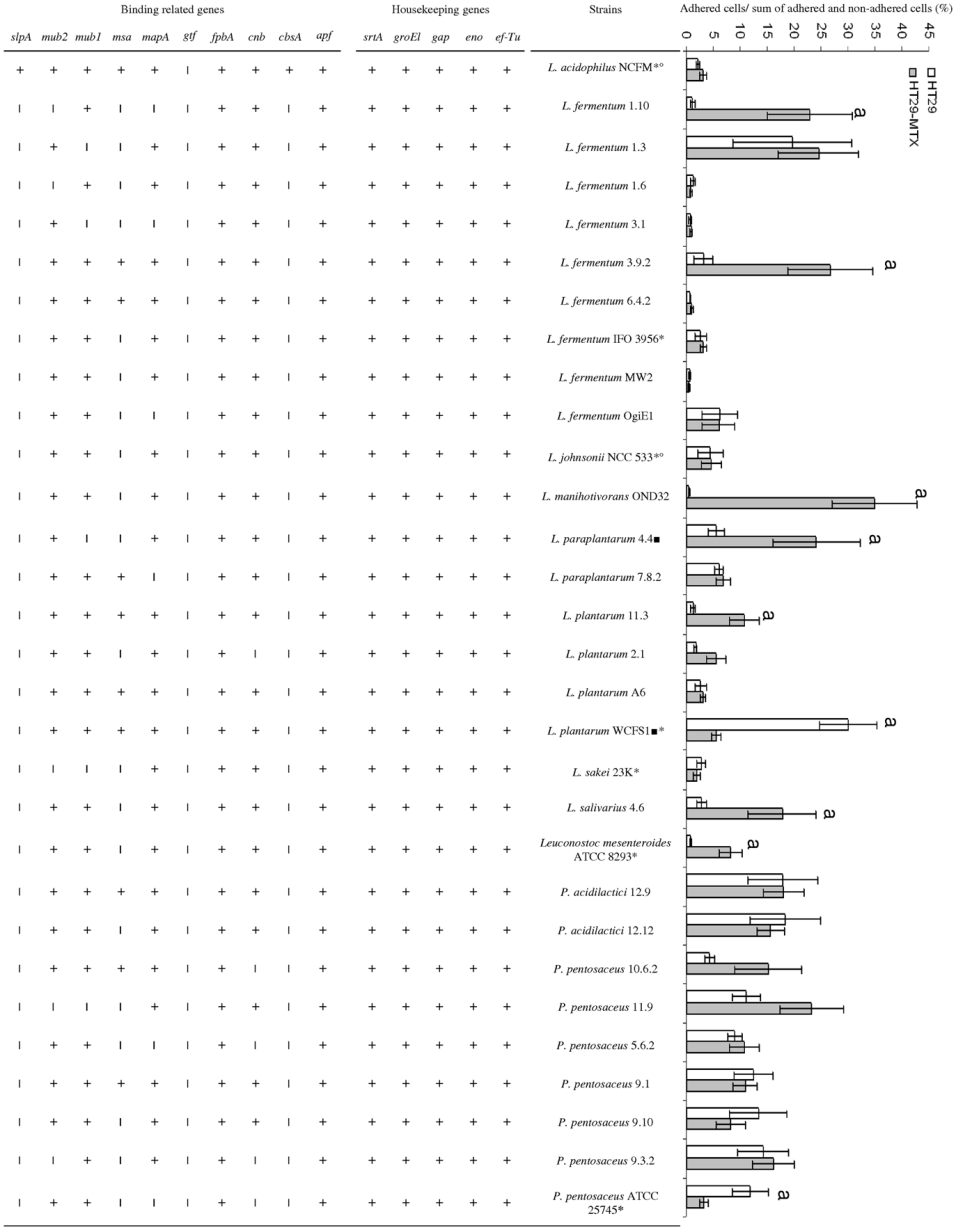
Ratio of adhered cells to the sum of adhered and non-adhered cells after 2 h incubation at 37°C and the distribution of genes involved in binding to the gastrointestinal tract in the 30 selected LAB. Results are the means ± SD of three independent assays. The ratio of bacteria bound to non-mucus secreting cells (HT29) is in white. The ratio of bacteria bound to mucus secreting cells (HT29-MTX) is in gray. The general role of the gene is indicated at the top left of the line. The absence of a gene is indicated by a “−” and its presence by a “+”. Asterisks indicate sequenced strains of LAB, circles indicate commercial probiotic strains, and squares the strains selected for transcript analysis. Letters indicate a statistical difference in the ratio between the two cell lines (p<0.05, Student-Newman-Keuls test).

Among the 163 *Lactobacillaceae* used in the study, we used a subset of 30 strains for the binding assays. The selection criteria were (i) bacteria belonging to each of the seven species that comprise the collection of LAB isolated from tropical amylaceous fermented foods (19 from pearl millet slurries and four from the other types of food); (ii) their genetic profiles were as dissimilar as possible; (iii) seven control strains were included in the analysis (Figure 2). Their ability to bind to mucus producing HT29-MTX cells and to non-mucus producing HT29 cells was evaluated. Assays on HT29 cells revealed high variability (0.6% to 30.0%) of binding properties among LAB, *L. plantarum* WCFS1 being the most efficient. The two well characterized strains, *L. johnsonii* NCC 533 and *L. acidophilus* NCFM, were able to bind to HT29 cells at a rate of 4.5% and 2.1% respectively and 11 LAB out of 19 isolated from the fermented pearl millet slurries showed higher binding ability than the reference probiotic *L. johnsonii* NCC 533 strains (5.0% to 19.6%). The other isolates had a lower binding capacity, similar to that of the control strains (0.7% to 4.3%). The *Pediococcus* genus (n = 9) showed higher binding ability than *Lactobacillus* (n = 20) with an average binding ability of 12.51%±1.4% versus 4.8%±1.6%, respectively.

When mucus secreting cells were used, the binding profile differed from the HT29 model (Figure 2) but there was still marked variation in binding ability between LAB (0.5% to 34.8%), *Lb. manihotivorans* OND32 being the most efficient strain. The *L. johnsonii* NCC 533 and *L. acidophilus* NCFM strains showed similar binding ability in the two cell models, and 16 LAB out of 19 isolated from the fermented pearl millet slurries showed higher binding ability than the two probiotic strains (5.6% to 26.7%). Like the HT29 model, *Pediococcus* tended to show higher binding ability to HT29-MTX cells than *Lactobacillus,* with an average binding capacity of 13.5%±2.0% versus 10.3%±2.4%, respectively. Strains isolated from tropical fermented foods showed higher binding ability than control strains.

Different behaviors were observed depending on the cell lines used. *L. fermentum* 1.10, *L. fermentum* 3.9.2, *L. manihotivorans* OND32, *L*. *paraplantarum* 4.4, *L. plantarum* 11.3, *L. salivarius* 4.6 and *Leuconostoc mesenteroides* ATCC 8293 displayed higher binding ability to HT29-MTX cells than to HT29 cells, while *L. plantarum* WCFS1 and *P. pentosaceus* ATCC 25745 bound more efficiently to HT29 cells than to HT29-MTX cells. The other LAB showed the same binding capacity whatever the cell models used.

### Mucin degradation and mucin utilization assays

**Figure 3:**
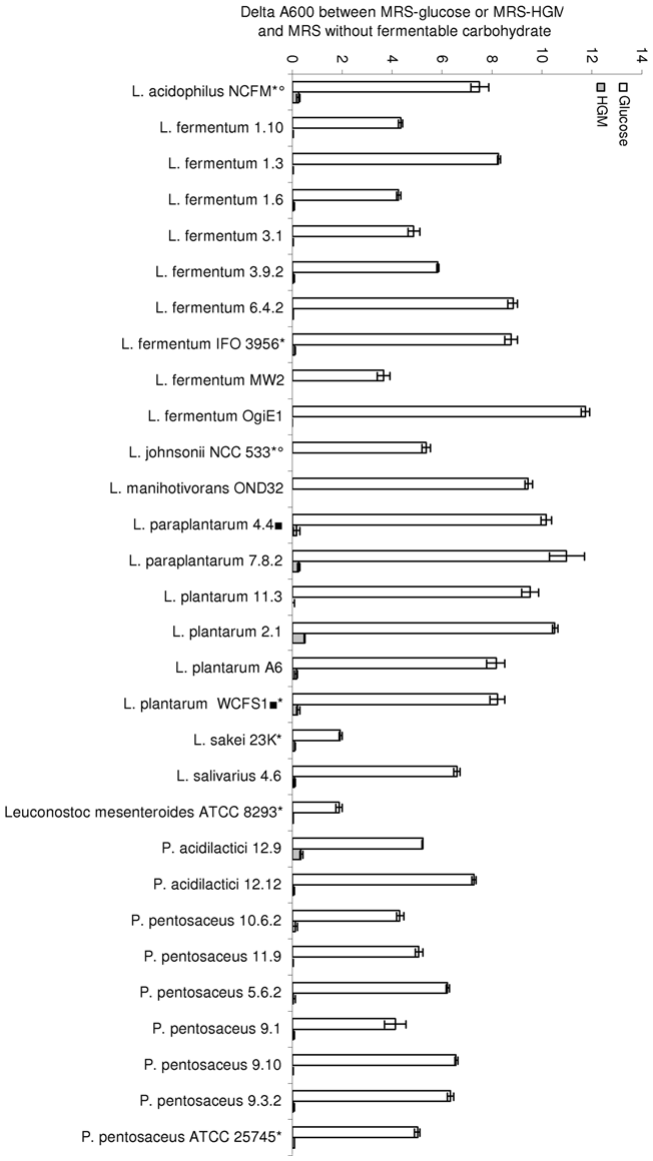
Delta A_600_ 24 h after inoculation of reconstituted MRS media containing 2.0% glucose (white) or 0.3% HGM (gray) and residual growth of LAB in MRS with no fermentable carbohydrate. Asterisks indicate sequenced strains of LAB, circles commercial probiotic strains, and squares the strains selected for transcript analysis.

To establish whether binding is linked with the ability to degrade or use mucin *in vitro*, degradation assays were conducted in solid and liquid media (Figure 3). No strain was able to degrade the glycoprotein of mucin, as evidenced by the absence of a mucin lysis zone in the Petri dishes. No growth or negligible growth was detected in all strains on the MRS medium containing mucin as sole fermentable carbohydrate.

### Expression of genes involved in the binding mechanism in bacteria

The expression of genes involved in binding was analyzed by measuring the mRNA in *L. paraplantarum* 4.4 and *L. plantarum* WCFS1, the two strains with different binding capacities in the two cell lines. *L. plantarum* WCFS1 bound better to HT29 cells than to HT29-MTX cells, and *L. paraplantarum* 4.4 exhibited an inverse phenotype.

**Figure 4:**
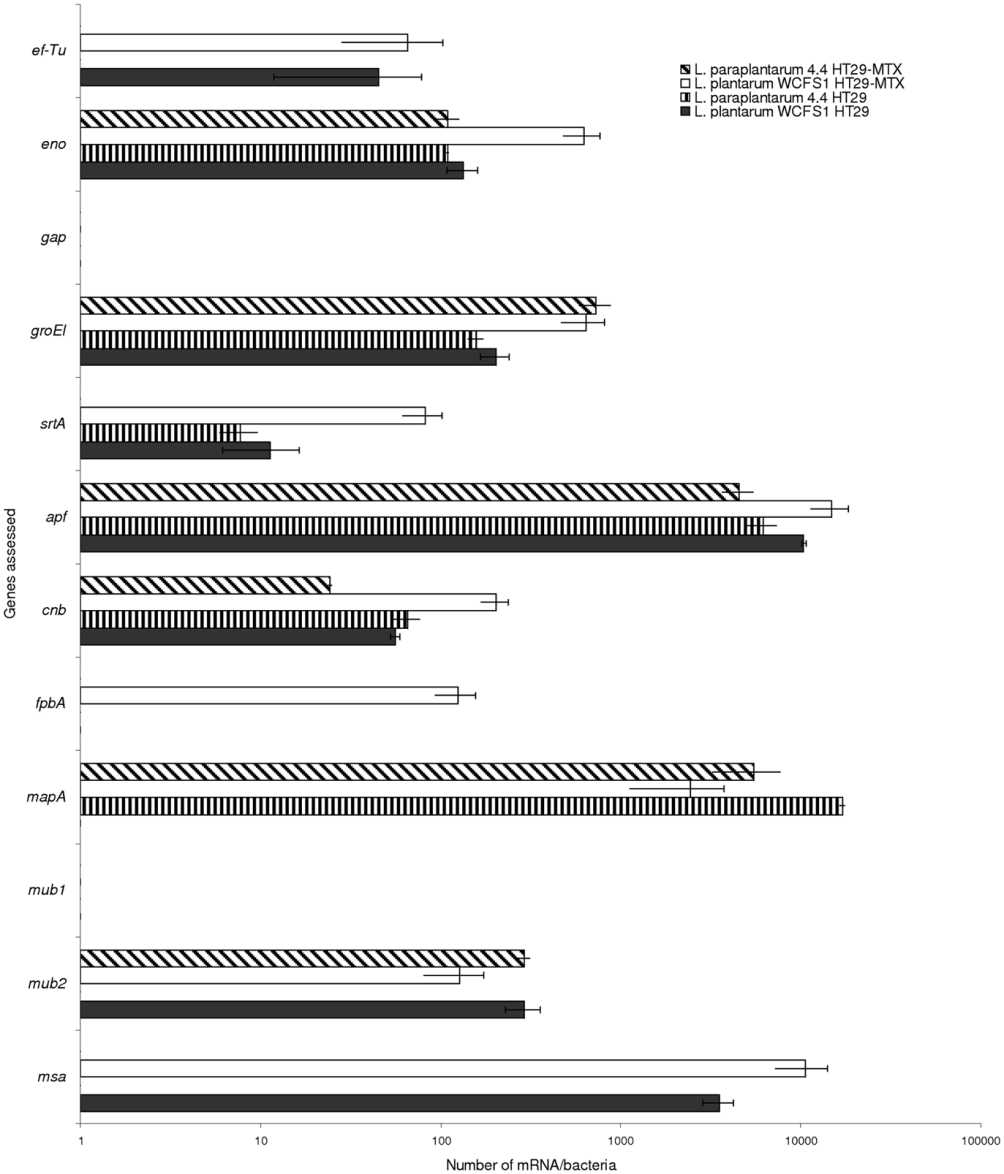
Copy number of mRNA/bacteria of binding related genes in *L. paraplantarum* 4.4 incubated with HT29 (diagonal hatched bar) or HT29-MTX (vertical hatched bar) and in *L. plantarum* WCFS1 incubated with HT29 (white) or HT29 MTX (black).

The genes *cbsA, gtf*, and *slpA* were not tested for their expression as they were not detected in these two isolates. The two strains expressed most of the genes involved in the binding process but with different profiles depending on the species and/or the cell model used (Figure 4). *L. plantarum* WCFS1 expressed *ef-Tu*, *eno, groEl, srtA, apf, cnb* and *mub2* genes when bound to HT29 cells. After contact with HT29-MTX cells, the strain also expressed *fpbA* and *mapA* genes. The transcripts of the genes *gap* and *mub1* were not detected whatever the cell line used. *L. paraplantarum* 4.4, which lacks the *mub1* gene, expressed the genes *eno*, *groEl, srtA, apf, cnb* and *mapA* when bound to HT29 cells. In the mucus secreting cells, the *srtA* gene transcript was no longer detected but *mub2* was expressed. The gene transcripts *ef-Tu, gap* and *fpbA* were not detected in either the HT29 or the HT29-MTX cell lines.

### Expression of *MUC2* genes in HT29 and HT29-MTX cell lines after contact with bacteria

**Figure 5:**
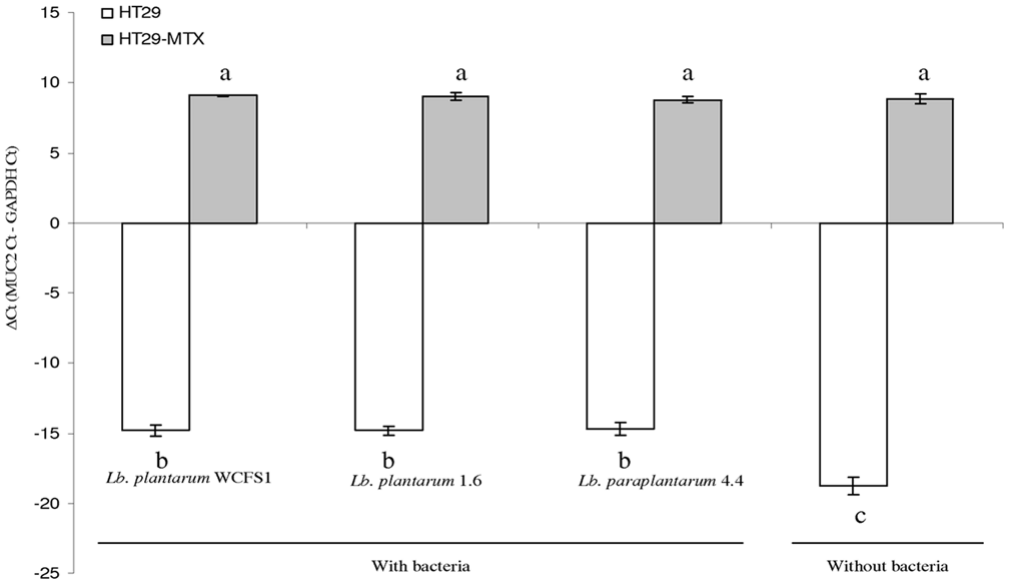
Expression of MUC2 in HT29 and HT29-MTX in response to cell binding bacteria. The delta Ct values of MUC2 normalized to the GAPDH gene obtained on HT29 is in white and on the HT29-MTX cell line is in gray. Different letters indicate a statistical difference between the samples (p<0.05, Student-Newman-Keuls test).

The expression of MUC2 genes was measured in HT29 and HT29-MTX cells after incubation for two hours in the absence of bacteria, or with three isolates that bound differently in the two cell models (Figure 2): *L. paraplantarum* 4.4, *L. plantarum* 1.6, and *L. plantarum* WCFS1 (Figure 5). The endogenous level of MUC2 is higher in HT29-MTX than in HT29 in the absence of bacteria. The presence or absence of bacteria did not influence the expression of MUC2 in HT29-MTX. In contrast, the HT29 cells displayed significantly higher MUC2 expression in the presence of bacteria than in their absence.

## Discussion

Our objective was to characterize the binding potential of a collection of 156 LAB isolated from traditional starchy fermented foods. One possible link between bacterial binding ability and genetic equipment was investigated in each LAB strain by comparing their corresponding gene set determined by PCR with their ability to adhere to enterocyte-like cells (HT29), and mucus secreting cells (HT29-MTX). To this end, genetic screening was used as it is expected to enable more rapid identification of any LAB potentially able to bind to the digestive tract than cell culture, which is more time consuming when used for a large set of bacterial isolates. Genetic screening has already been used by other teams to select potentially adhesive probiotic strains of *L. plantarum*, but with fewer target genes (*msa, mub*, and *fpbA*) and only one species [15], [16] than in our study, which included 14 binding related genes in strains belonging to seven different species. We previously used the same strategy to estimate the potential of other nutritional or probiotic characteristics in the same bacterial collection [13].

### Adhesion varied considerably among the isolates

The adhesion tests performed on a selected subset of 30 LAB revealed different binding capacities ranging from 0.6% to 30.0% on the HT29 cells and from 0.5% to 34.8% on the mucus producing cell line HT29-MTX. Such variability in the binding ability of *Lactobacillus* strains to mucus and lectin was also recently observed in *L. casei* and *L. reuteri* species [17], [18]. Most of the 23 strains isolated from amylaceous fermented foods showed higher binding ability than the two probiotic candidate strains (*L. acidophilus* NCFM and *L*. *johnsonii* NCC 533). This was particularly true for *L. manihotivorans* OND32 and for some *L. fermentum* strains in HT29-MTX and of the *Pediococcus* genus in both cell models. A similar result was found for *L. plantarum* Lp9, which exhibited a higher adhesion ratio to a non-mucus secreting cell line [16] than the two probiotic strains used here. Nevertheless, it would be interesting to compare a larger number of wild and probiotic strains. However, the strains from starchy foods are good candidates for further investigation of their use as probiotics for the sustainable production of beneficial molecules such as vitamin B, since they display a high genetic potential for their synthesis [13].

### Some genes have similar presence or absence rates in different isolates

As expected, the five housekeeping genes (*gap*, *ef-tu*, *eno*, *groel,* and *srtA*) were found in all the isolates, so it was not necessary to screen them to determine the binding potential of the bacteria although they could to some extent be considered as positive controls for gene detection. Among the other genes found in the entire bacterial collection, the non-essential gene *fpbA,* which codes for a fibronectin binding protein, has been reported to be present in a range of pathogenic species. Analysis of its sequence alignment showed that it is present in numerous LAB species [19], [20]. This suggests that LAB and pathogens may share similar binding mechanisms involving proteins with similar functions, confirming the observation that some LAB are able to inhibit pathogen adhesion to intestinal cells simply through competition [21]. No large conserved domains were identified in the aggregation promoting protein LBA0493, but the wide distribution of the corresponding *apf* gene among *Lactobacillus* species and the existence of a small conserved sequence mainly confined to the C-terminal region of the protein [22] could explain the detection of this gene in all the bacteria. Conversely, sequence alignment analysis of the *cbsA* and *slpA* genes showed that they are particularly distributed in *L. acidophilus* and *L. brevis* phylogenetic groups, explaining why they were not detected in our bacterial collection. As *gap*, *ef-tu*, *eno*, *groel, srtA, apf, cbsA, fpbA, slpA* genes had also been found in the bacterial collection, their detection was not required to determine an adhesion ratio in the *Lactobacillaceace* species concerned.

### Genetic profiles did not appear to be linked to binding capacity

The different binding abilities of the 30 selected LAB cannot be explained by their genetic profile. Variability of gene detection was found in only five genes in the bacterial collection. Among the most widely represented genes, *cnb*, *mapA, mub1*, and *mub2* genes were detected in 94.5%, 86.5%, 96.5%, and 95.5% of the strains, respectively. The gene coding for mucus binding proteins has already been shown to be involved in adhesion to HT29 cells, Caco-2 cells, mucus and mucin in *L. reuteri* 1063, *L. acidophilus* NCFM, and *L. salivarius* UCC118 [23], [24], [25]. The *msa* and *mub* genes also contain MucBP domains. However the *msa* gene was the gene related to binding that was detected the least frequently in our collection. Its detection rate (20%) in our *L. plantarum* isolates was even lower than the rate (40%) reported in other strains of *L. plantarum* [15]. This could be explained by the high variability of nucleic sequences due to large deleted sequences found in this gene among *L. plantarum* strains [26], [27]. Even though several sequences were selected to design the corresponding primer set, it can be hypothesized that, in some cases, this primer failed to detect *msa* if the targeted sequence corresponded to a deleted region in some *L. plantarum* genes. This could have led to underestimation of the presence of this gene among the LAB genomes tested in our collection.

In 21 LAB with different genetic equipment, adhesion was similar in the two cell models, suggesting that the mucus layer did not influence binding and that there was no link with the genetic equipment. In nine LAB, the mucus layer appeared to play a critical role in the binding mechanism. Indeed, in seven LAB, binding to mucus secreting cells was more efficient, whereas binding to HT29 cells was more efficient in the two remaining strains. However, no genes were found to be linked to a binding property in a particular cell line. It is possible that differences between the LAB are due to newly described genes involved in binding functions such as *spa* genes [28], *mbf* [29], *mcrA* [30], *mabA* [31], *lam29* [32], *p40* [33], or *cbp* [34] that were not included in this study because they were published after the completion of this work.

### Adhesion is not linked to mucus utilization

Mucins are the major structural components of the mucus found in the gastrointestinal tract and it is widely accepted that they control the growth of commensal bacteria [35], [36]. The binding ability of LAB to mucus may give them an ecological advantage through easier interaction with glycoproteins in the mucus and their utilization. Nonetheless, none of the 30 LAB tested was able to grow with a commercial gastric mucin as sole fermentable carbon source, or to degrade the protein of the same mucin. Utilization of mucus thus cannot explain the different binding abilities of the 30 selected LAB. A previous study showed that mucin degradation in *Lactobacillaceae* species is not widespread but some strains belonging to *L. mucosae* species, which is prevalent in the short bowel syndrome in humans [37], demonstrated this ability *in vitro* [38], [39]. The mucus degradation capacity is controversial. Indeed, mucus has protective functions but its degradation by bacteria has been recognized to be involved in mucin regulation and turnover and hence to contribute to intestinal integrity [38].

### MUC2 expression by eukaryotic cells is not linked to binding of *Lactobacillus*

We also checked if binding ability was linked to tighter cross-talk between bacteria and eukaryotic cells by measuring the expression of the gel forming gene MUC2. Strains *L. plantarum* WCFS1, *L. fermentum* 1.6 and *L. paraplantarum* 4.4, which have quite different binding phenotypes, were all able to induce the expression of this gene after two hours of incubation with HT29. Similar observations have previously been reported for different probiotics [40], [41], [42]. No such induction was observed with HT29-MTX cells for any strain. Due to methotrexate treatment, HT29-MTX are known to express a high level of MUC2 without bacteria, and this could explain why a modulation of the expression of MUC2 genes was not detected in presence of the bacteria [43]. However previous studies showed that MUC2 expression can still be modified in the HT29-MTX cell line in response to infection by *Escherichia coli* [44]. The expression of MUC2 does not appear to be linked with the actual binding capacity of the strains, suggesting a different induction mechanism is involved [45].

### Measurement of gene expression *vs.* gene detection

Transcriptomic analysis of LAB adaptation to a specific environment or stress has been widely used to investigate important genes involved in this adaptation [46], [47], [48]. To our knowledge, gene expression of LAB bound to cell models is not frequently reported in the literature [49], [50]. The difference in binding capacity between LAB strains could also be due to the differential expression of binding related genes. It is thus important to bear in mind that genetic screening has its own limitations due to possible false positives, such as amplification of pseudogenes by PCR, or false negatives due to nucleic sequence variability, like for the *msa* gene. As a consequence, with our strategy, the existence of mutations cannot be excluded, leading to the detection of inactive genes like the *fpbA* gene that were detected by PCR in *L. paraplantarum* 4.4, but not expressed in HT29 or HT29-MTX cells. However, the expression of most of the genes screened in *L. plantarum* WCFS1 and *L. paraplantarum* 4.4, which displayed different binding capacities in the adhesion tests, varied with the strain and with the cell line concerned. Such a difference was previously observed in some proteins involved in the binding function in other *L. plantarum* strains with different binding ability to mucus [51]. In this study, the transcripts of *gap* and *mub1* were not detected in *L. plantarum* WCFS1 nor was *gap* detected in *L. paraplantarum* 4.4 (which lacks *mub1* and *msa*) suggesting that neither gene plays an important role in binding to these cell lines for these strains. This was surprising since *gap* is an essential gene. As the LAB was incubated in complete cell media not favorable for LAB growth, presumably the level of transcripts of *gap* genes was not sufficient to be detected in these two strains. Indeed, a previous study showed that GAPDH is only overexpressed in highly adhesive strains of *L. plantarum* in the presence of mucus [51]. However other housekeeping genes *eno*, *groEl* but also binding related genes *apf* and *cnb*, were expressed in both strains when bound to both cell models, but as *eno* and *groEl* are housekeeping genes, it is possible that *apf* and *cnb* play a more important role in cell binding. The *fpbA*, *srtA*, *mapA* and *mub2* genes were expressed differently depending on the bacteria and the cell line. However no link was found between the expression of these genes and the binding ability of the LAB we tested, despite previous works that identified the functional role of each of these genes in cell binding [25], [52]. For instance, in strain WCFS1, which bound better to HT29 cells, *mapA* was only induced in HT29-MTX cells, whereas in both cell models, it was induced in *L. paraplantarum* 4.4, which bound more tightly to HT29-MTX cells. In contrast, *srtA* was induced in both cell lines in strain WCFS1 whereas it was only induced in HT29 cells in *L. paraplantarum* 4.4. And finally, *mub2* was only expressed in *L. paraplantarum* 4.4 in HT29-MTX cells, whereas it was expressed in both cell lines in the lower binding strain WCFS1. These results suggest that the cell type influences gene expression, which varies depending on the LAB strain concerned. In this regard, measurement of gene expression is more informative than gene detection. However it could not be directly linked to binding ability, suggesting that more specific markers, if any, need to be investigated.

In conclusion, genetic screening provided the opportunity to evaluate the distribution of genes known to be involved in cell binding in both wild isolates and reference strains. It could have been an ideal tool to assess potential bacterial adhesion, but proved to be inadequate, since there was a gap between the potential identified by screening and the results obtained by functional analysis. The importance of the mucus layer in the binding mechanism was highlighted in many strains, since different adhesion patterns were obtained depending on whether mucus was produced or not. This analysis also showed that wild LAB from tropical amylaceous fermented food have a much higher binding capacity than two LAB currently recognized to be probiotics. These food niches could be a source of new probiotics and thus deserve more detailed investigations of their properties. Although many strains were shown to possess the target genes, we still need to improve our understanding of how these genes are regulated in relation with the cell models used and during the passage of the bacteria through the gastrointestinal tract, and also to evaluate the functionality of the corresponding enzymes in this environment.

## Materials and Methods

### Bacteria and culture conditions

Bacterial isolates were routinely cultured at 30°C in de Man, Rogosa and Sharpe (MRS) broth (Difco, Le Pont de Claix, France). The LAB used in this study came from our collection which consists of isolates (n = 152) from fermented pearl millet slurries sampled in traditional production units in Ouagadougou (Burkina Faso). This collection is composed of LAB belonging to the genus *Pediococcus* (*P. pentosaceus*, *P. acidilactici*) and *Lactobacillus* (*L. fermentum*, *L*. *paraplantarum*, *L. plantarum*, and *L. salivarius*) (Figure 1). LAB from other fermented foods and probiotic strains were also used. *L. plantarum* A6 (LMG 18053) [53], *L. fermentum* Ogi E1 (CNCM I–2028) and *L. fermentum* MW2 (CNCM I–2029) [54], *L. manihotivorans* OND32 [55] were from different tropical starchy fermented foods; *L. sakei* 23K [56] was sampled from French sausage and *L. johnsonii* NCC 533 [57] and *L. acidophilus* NCFM [58] were probiotic strains. The control strains used for gene screening were *P. pentosaceus* ATCC 25745 [14], *Leuconostoc mesenteroides* ATCC 8293 [14], *L. plantarum* WCFS1 [59], *L. fermentum* IFO 3956 [60], and *L. acidophilus* NCFM [58].

### DNA extraction

DNA was extracted from the bacterial pellet of overnight pure cultures using the Wizard genomic DNA purification kit (Promega, Charbonnières, France) with an additional lysis step using an amalgamator with zirconium beads (VWR, Fontenay-sous-Bois, France).

### Primer design

Genetic screening was based on a set of genes involved in the binding mechanism. These genes are listed in Table 1. To detect their presence, the DNA extracted from the isolates was screened by PCR amplification. The primers for each PCR reaction were designed by comparing sequences resulting from functional analysis with the genomic and protein database (NCBI) using BLASTn, BLASTp and BLASTx algorithms (as of April 2009). This analysis was mainly limited to species present in our bacterial collection. Once selected, nucleotide sequences were aligned using the clustalW program [61] to generate a single consensus sequence [62] that was exploited to design the primers using primer3 software [63]. All primers were synthesized by Eurogentec (Angers, France).

### PCR amplification for the detection of binding-related genes

Each 20-µl PCR mixture contained a reaction cocktail of 200 µM (each) of deoxynucleoside triphosphate, 0.5 µM of each primer, 3.5 mM of MgCl_2_, 0.5 U of *Taq* DNA polymerase (Promega), 10X taq buffer and 150 ng of DNA template. The PCR conditions were one cycle at 95°C for 5 min, 40 cycles at 95°C for 30 s, at annealing temperature (for 10 s) depending on the primer used (Table 1), and at 72°C for 15 s, followed by one cycle at 72°C for 5 min using the thermal cycler (Applied Biosystems Veriti™ VWR, Strasbourg, France). The PCR products were separated on agarose gel and then stained with ethidium bromide to check for the presence of a single amplicon. When a gene from a species was amplified using a primer initially designed for a different species, the corresponding amplicon was sequenced (MWG Operon, Germany).

### Cell culture

The HT29 revG- and HT29-MTX cells lines were used between the 58^th^ to 63^rd^ and the 20^th^ to 25^th^ passage respectively. Mucus secreting HT29-MTX cells were obtained from Thecla Lessuffleur (INSERM UMR S 938, Paris, France) [43]. Cells were routinely grown in Dulbecco's modified Eagle's minimal essential medium (DMEM) with 4.5 g/L glucose (Lonza, Verviers, Belgium), supplemented with 10% (v/v) fetal calf serum (FCS) inactivated for one hour at 56°C (Lonza, Verviers, Belgium), with 1% (v/v) L-Glutamine 200 mM (Lonza, Verviers, Belgium), and 1% (v/v) penicillin-streptomycin (Lonza, Verviers, Belgium). Monolayers of both cells lines were prepared in six-well tissue culture plates and inoculated at a concentration of 10 10^4^ and 12 10^4^ cells per ml for HT29 and HT29-MTX, respectively. Fully differentiated cells were obtained 21 days after plating. Two days before the adhesion assay, antibiotics were no longer used in the cell cultivation media. All experiments were carried out at 37°C and cells were maintained in a 10% CO_2_:90% air atmosphere at the same temperature. The culture medium was changed daily.

### Adhesion assay

The adhesion assay was performed on a subset of 30 LAB selected as controls, or harboring different genetic equipment and belonging to different species. Overnight cultures of bacteria grown in MRS at 30°C were centrifuged for 10 min at 8 000×g. The pellet was re-suspended in complete DMEM without antibiotics at a final concentration of 10^7^ CFU/ml and was then incubated for 24 hours at 37°C. The pellets were then centrifuged for 10 min at 8 000×g, washed twice with phosphate-buffered saline (PBS) pH 7, 37°C (Lonza, Verviers, Belgium), and re-suspended in complete DMEM, at 37°C without antibiotics. Initial viable bacteria were counted by plating on MRS agar. Before the adhesion assay, the HT29 and HT29-MTX cells were gently washed twice with sterile PBS at pH 7 at 37°C (Lonza, Verviers, Belgium). The bacterial suspension was added to each well of the cell line (with a bacterial cell to epithelial cell ratio of ∼10∶1), and incubated in a 10% CO_2_:90% air atmosphere at 37°C for 2 h. After incubation, the viability of non-adherent bacteria from the supernatants was determined by plating serial dilutions on MRS agar. The HT29 and HT29-MTX monolayers were gently washed four times with PBS to remove unattached bacteria. Cell monolayers were scraped with 0.1% (v/v) Triton® X-100 (Sigma), and passed twice through a 21×g needle and then incubated for 30 min at room temperature. Appropriate dilutions were plated on MRS agar. The results of the adhesion assay were expressed as an adhesion percentage, i.e. the ratio of adherent bacteria to the total number of bacteria added to each well. Three independent experiments (n = 3) were performed, with two replicates of each experiment.

### Total RNA extraction and reverse transcription

Three isolates were selected based on their different binding capacities and incubated in the same conditions as described in the previous paragraph except that cells were grown in 60 cm^2^ Petri dishes. All experiments were performed in triplicate. The washed monolayers were scratched with TE buffer (1 mM EDTA, 10 mM Tris, pH 7, Promega) and the resulting suspension was lysed in a Tissue Lyser (Qiagen, Germany) in acid phenol at pH 4 (Eurobio, Ulysse, France) with zirconium beads (VWR, Fontenay-sous-Bois, France) to allow disruption of cells and bacteria. After centrifugation, the aqueous phase was transferred in TRIzol® Reagent (Invitrogen, Carlsbad, USA) and incubated for 5 min at room temperature. After addition of chloroform (Carlo Erba, Val de Reuil, France), the solution was centrifuged at 10000×g for 15 min) and the nucleic acid was precipitated by the addition of isopropanol (Sigma, St Louis, USA). The pellet was washed in 70% ethanol (Carlo Erba, Val de Reuil, France), suspended in nuclease free water (Promega, Madison, USA), and kept overnight at −80°C. The quality of the RNA was checked using NanoDrop ND-1000 (Thermo Scientific, Illkirch, France) and Bioanalyzer 2100 (Agilent technologies, Massy, France) at the PICT platform, INRA, Jouy-en-Josas, France. The DNA was removed with RQ1 RNase-Free DNase (Promega, Charbonnières, France) and the cDNA was obtained using the Reverse Transcription System (Promega, Charbonnières, France) following the manufacturer's instructions. The absence of genomic DNA in treated RNA samples was checked by semi-quantitative PCR using the following primers: 338f converted into its reverse complement, 5′ CTGCTGCCTCCCGTAGGAGT 3′ [64] and Lpla72f, 5′ ATCATGATTTACATTTGAGTG 3′ [65] specific to the 16 S rRNA gene sequence of *L. plantarum*. For treated eukaryotic RNA samples, the absence of genomic DNA was checked by semi-quantitative PCR using the primers hGAPDH: 5′ TGACGCTGGGGCTGGCATTG 3′ and 5′ GGCTGGTGGTCCAGGGGTCT 3′ [66].

### Semi-quantitative PCR

All measurements were performed in duplicate using the QPCR system (Stratagene, Mx3005p™) and Syber Green technology (Eurogentec, Angers, France). For each reaction, 1 µL of the cDNA template was added to 15 µL of PCR mix containing 1X MESA GREEN qPCR MasterMix Plus (Eurogentec, Angers, France) and 0.3 µM of each primer. The PCR conditions used were 10 min at 95°C and 40 cycles of 30 s at 95°C, then 30 s at 50°C, then 30 s at 72°C, followed by a dissociation gradient from 55°C to 95°C. For bacterial gene expression, the cDNA of the 16S rRNA was determined in parallel for each sample using the 518r and Lpla72f primer set. Absolute quantification of the 16S rRNA copy number was done using a standard curve method based on known bacterial concentrations. For eukaryotic gene expression, GAPDH was used as the reference gene and the hMUC2 primers were used for MUC2 quantification: 5′ GGGGACAGTGGCTGCGTTCC 3′ and 5′ CGGGGCAGGGCAGGTCTTTG 3′ [66]. Results obtained on MUC2 were normalized using the following formula: fold change  =  ΔCt, where the ΔCt threshold cycle (Ct) equals (MUC2 Ct – GAPDH Ct) of the sample. Data were analyzed using MxPro QPCR software 2007 Stratagene version 4.10. Table 1 shows the efficiency of the real time PCR assays for each primer.

### Mucin assay

The ability of isolates to degrade mucin was evaluated by measuring the mucin lysis zone on plate assays as previously described, with some minor modifications [38], [67]. Briefly, glucose (20.00 g/l, Sigma, St Louis, USA) or hog gastric mucin type III (3.00 g/l, Sigma) were incorporated in reconstituted MRS (10.00 g/l proteose peptone, 10.00 g/l beef extract, 5.00 g/l yeast extract, 2.00 g/l ammonium citrate, 5.00 g/l sodium acetate, 0.10 g/l magnesium sulfate, 0.05 g/l manganese sulfate, and 1.00 g/l Tween 80, and 2.00 g/l dipotassium phosphate, Becton Dickinson, Le Pont-De-Claix, France). Five microliters of overnight bacterial cultures were spotted onto the surface of the agar medium in a Petri dish. The plates were incubated at 37°C without shaking for 72 h and then stained with Amido black (3 g/l, RAL, Martillac, France) in acetic acid (3.5 M, Sigma) for 30 min. The plates were then washed with acetic acid (1.2 M, Sigma) until the mucin lysis zone (discolored halo) appeared around the positive control cultures (human fecal flora, diluted 100 times). The mucin degradation activity was defined by the size of the mucin lysis zone.

The ability of our isolates to grow in the presence of mucin was tested in liquid cultures as previously described, with some minor modifications [68]. Briefly, the growth of isolates in reconstituted MRS with glucose 20.0 g/l or with hog gastric mucin 3.0 g/l was monitored by measuring *A*_600_ after 2% (v/v) inoculation and 24 hours after incubation at 37°C without shaking. The results are expressed as *A*_600_ obtained 24 hours after inoculation of reconstituted MRS media containing 20.0 g/l glucose or 3.0 g/l HGM minus the residual growth of LAB obtained in reconstituted MRS media containing no fermentable carbohydrate.
